# Del-1 Plays a Protective Role against COPD Development by Inhibiting Inflammation and Apoptosis

**DOI:** 10.3390/ijms25041955

**Published:** 2024-02-06

**Authors:** Nakwon Kwak, Kyoung-Hee Lee, Jisu Woo, Jiyeon Kim, Jimyung Park, Chang-Hoon Lee, Chul-Gyu Yoo

**Affiliations:** 1 Division of Pulmonary and Critical Care Medicine, Department of Internal Medicine, Seoul National University Hospital, Seoul 03080, Republic of Korea; 2 Department of Internal Medicine, Seoul National University College of Medicine, Seoul 03080, Republic of Korea

**Keywords:** COPD, Del-1, inflammation, apoptosis, Nrf2

## Abstract

Neutrophilic inflammation is a prominent feature of chronic obstructive pulmonary disease (COPD). Developmental endothelial locus-1 (Del-1) has been reported to limit excessive neutrophilic inflammation by inhibiting neutrophil adhesion to the vascular endothelial cells. However, the effects of Del-1 in COPD are not known. We investigated the role of Del-1 in the pathogenesis of COPD. Del-1 protein expression was decreased in the lungs of COPD patients, especially in epithelial cells and alveolar macrophages. In contrast to human lung tissue, Del-1 expression was upregulated in lung tissue from mice treated with cigarette smoke extracts (CSE). Overexpression of Del-1 significantly suppressed IL-8 release and apoptosis in CSE-treated epithelial cells. In contrast, knockdown of Del-1 enhanced IL-8 release and apoptosis. In macrophages, overexpression of Del-1 significantly suppressed inflammatory cytokine release, and knockdown of Del-1 enhanced it. This anti-inflammatory effect was mediated by inhibiting the phosphorylation and acetylation of NF-κB p65. Nuclear factor erythroid 2-related factor 2 (Nrf2) activators, such as quercetin, resveratrol, and sulforaphane, increased Del-1 in both cell types. These results suggest that Del-1, mediated by Nrf2, plays a protective role against the pathogenesis of COPD, at least in part through anti-inflammatory and anti-apoptotic effects.

## 1. Introduction

Chronic obstructive pulmonary disease (COPD), characterized by persistent respiratory symptoms and airflow limitation, is the third leading cause of morbidity and mortality worldwide [1]. The estimated prevalence of COPD is 10.9% and approximately three million people die each year from COPD [2,3]. The main cause of COPD is long-term exposure to harmful particles or gases, including cigarette smoke, biomass fuels, and air pollution [4]. These trigger the release of inflammatory mediators by lung epithelial cells. In response to the inflammatory signals, immune cells, such as neutrophils, macrophages, and cytotoxic T lymphocytes, are recruited to the lungs [5]. Of these, neutrophilic inflammation is a prominent feature of COPD. Neutrophilic inflammation leads protease/anti-protease imbalance, apoptosis, and cellular senescence, and ultimately the development of COPD [6]. In addition to the development of COPD, neutrophilic inflammation is also involved in the acute exacerbation of COPD (AE-COPD), which is associated with increased morbidity and mortality [7]. 

Developmental endothelial locus-1 (Del-1), a 52-kDa glycoprotein, consists of three N-terminal epidermal growth factor-like repeats and two C-terminal discoidin I-like domains. Del-1 exists in two forms—an extracellular soluble form and an intracytoplasmic form. Endothelial cell-derived extracellular soluble Del-1 inhibits interactions between β2 integrins on neutrophils (lymphocyte function-associated antigen-1) and their counter receptors on endothelial cells (intracellular adhesion molecule-1), thereby preventing neutrophil adhesion and extravasation. Thus, extracellular soluble Del-1 inhibits the initiation of neutrophilic inflammation by inhibiting neutrophil recruitment [8]. 

In addition to regulating neutrophil recruitment, extracellular soluble Del-1 modulates inflammatory responses in several ways. It counteracts IL-17 production [9], binds to apoptotic neutrophils, and enhances their clearance via efferocytosis [10], thereby reducing the inflammation. In addition, macrophage-derived extracellular soluble Del-1 promotes the upregulation of TGF-β and resolvins [9]. Therefore, extracellular soluble Del-1 may contribute to the restoration of tissue integrity and homeostasis through its anti-inflammatory effects. In contrast, little is known about how Del-1 functions at the intracellular rather than the extracellular level.

Previously, we have shown that plasma levels of Del-1 are lower in patients with COPD and that this reduced Del-1 level predicts the subsequent risk of AE-COPD [11]. These findings suggest that extracellular soluble Del-1 plays a protective role against the development of both COPD and AE-COPD. Based on these findings, we hypothesized that Del-1 is also involved in the development of COPD, and investigated the role of Del-1 in the pathogenesis of COPD and its underlying molecular mechanisms.

## 2. Results

### 2.1. The Expression Level of Del-1 in Human Lung Tissues

**Figure 1:**
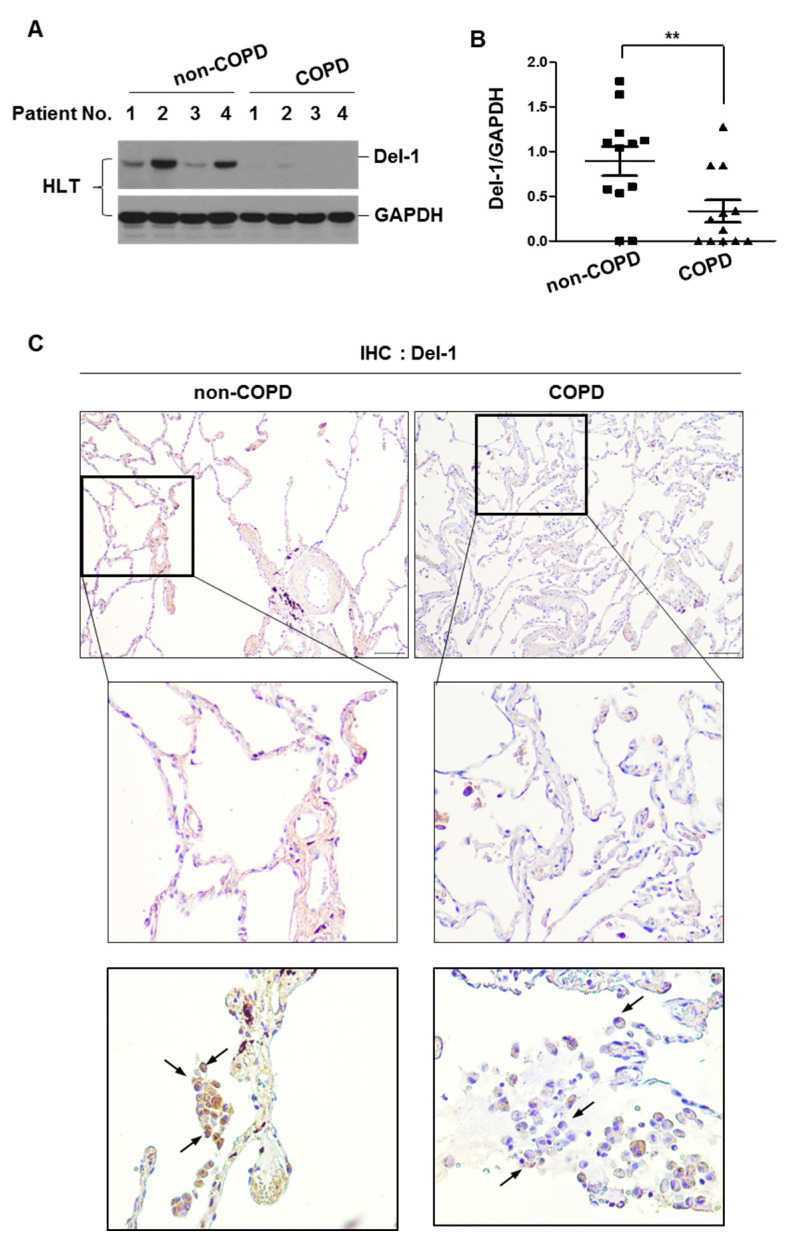
The expression level of Del-1 in human lung tissues. (**A**,**B**) Lung lysates from non-COPD (smokers without emphysema, n = 12) and COPD patients (smokers with emphysema, n = 12) were subjected to Western blot analysis for Del-1 and GAPDH. (**B**) Gel data were quantified using Scion image software Version 4.0. Data represent mean ± SE. ** *p* < 0.05. (**C**) Del-1 immunohistochemistry of lung tissues from non-COPD (smokers without emphysema, n = 6) and COPD patients (smokers with emphysema, n = 5). Arrows indicate the macrophages. Original magnifications, ×100 and ×200. HLT, human lung tissue; IHC, immunohistochemistry.

To investigate the role of Del-1 in COPD development, we first measured the level of Del-1 protein expression in surgically resected human lung tissues isolated from non-COPD (smokers without emphysema, n = 12) and COPD patients (smokers with emphysema, n = 12). Del-1 expression was lower in the lung homogenates of patients with COPD than in the lung homogenates of non-COPD patients (Figure 1A,B). Immunohistochemical staining showed that decreased Del-1 was prominent in epithelial cells and immune cells such as macrophages (Figure 1C). 

### 2.2. The Expression Level of Del-1 in Lungs of CSE-Instilled Mice and Its Impact on Oxidative Stress

**Figure 2:**
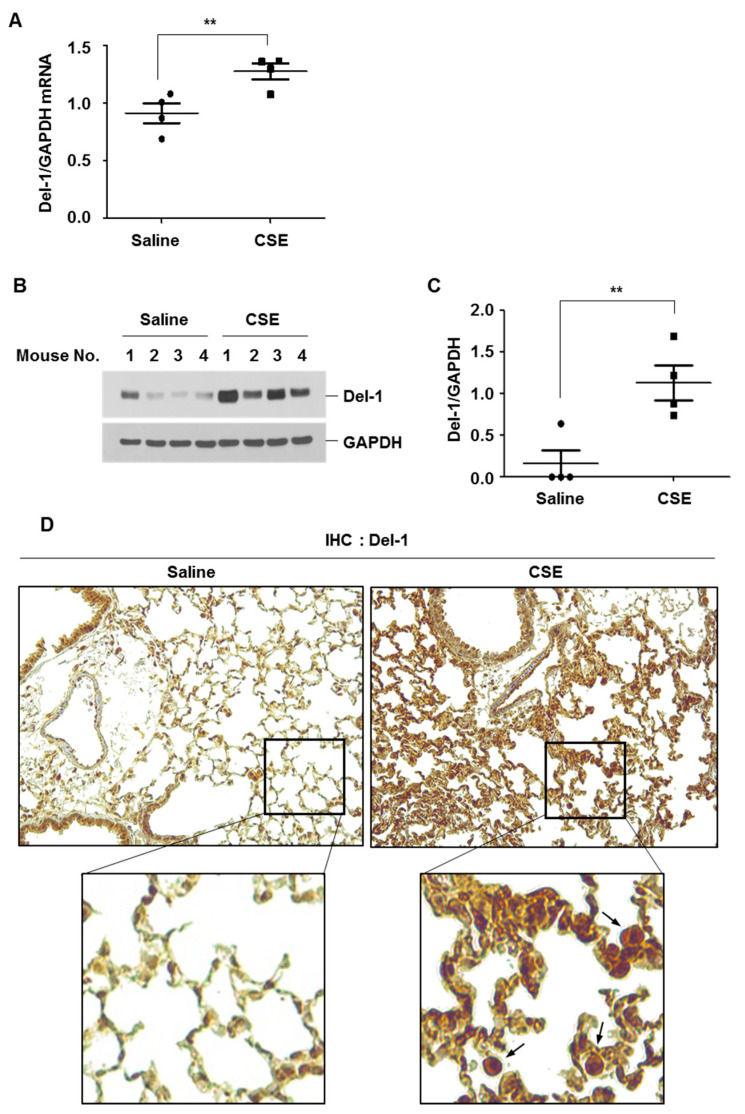
Intratracheal CSE instillation upregulated the expression levels of *Del-1* mRNA and protein in lung tissues of C57BL/6 mice. (**A**–**C**) C57BL/6 mice were intratracheally instilled with saline or CSE, as described in the Materials and Methods section (saline n = 4, CSE n = 4). Mice were sacrificed at day 50 after the first instillation, to isolate lungs. (**A**) Total RNA was extracted, and quantitative real-time PCR for *Del-1* and *GAPDH* was performed. Data represent mean ± SE. ** *p* < 0.05. (**B**) Lung lysates from mice were subjected to Western blot analysis for Del-1 and GAPDH. (**C**) Gel data were quantified using Scion image software Version 4.0. Data represent mean ± SE. ** *p* < 0.05. (**D**) Del-1 immunohistochemistry of lung tissue samples from saline- or CSE-treated mice. Arrows indicate the macrophages. Original magnifications, ×100 and ×200.

In our previous report, we demonstrated that intratracheal instillation of CSE induced inflammation and emphysema in the lungs of C57BL/6 mice [12]. In the current study, we examined the expression levels of Del-1 in the lung tissues of CSE-instilled mice. CSE instillation led to an increase in Del-1 mRNA and protein levels in lung tissues (Figure 2A–C). Similarly to human lung tissues, Del-1 expression was predominantly observed in the lung epithelial cells and immune cells, particularly the alveolar macrophages (Figure 2D). 

We previously demonstrated that intratracheal instillation of CSE induced more severe emphysema in Del-1 KO mice than in WT mice [11]. In the lung tissues of Del-1 KO mice, the surrogate markers of oxidative stress (8-OHdG and 4-HNE) were increased (Supplementary Figure S1), suggesting the protective effects of Del-1 against COPD development via an anti-oxidative mechanism.

### 2.3. Del-1 Suppressed CSE-Induced IL-8 Production and Apoptosis in Lung Epithelial Cells

**Figure 3:**
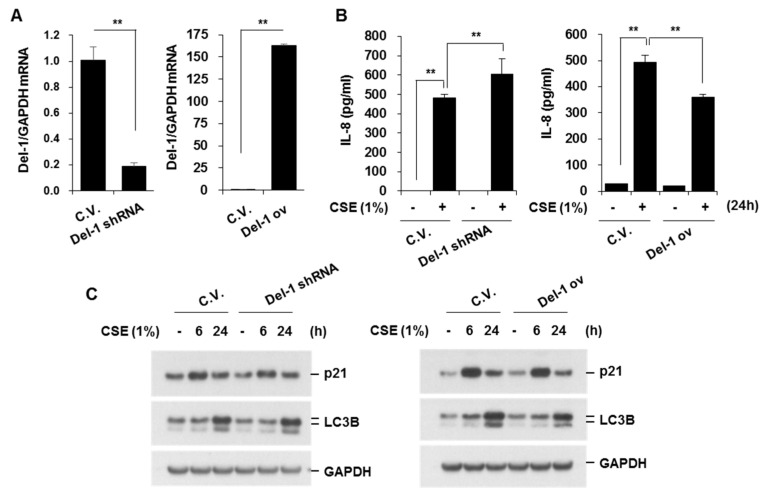

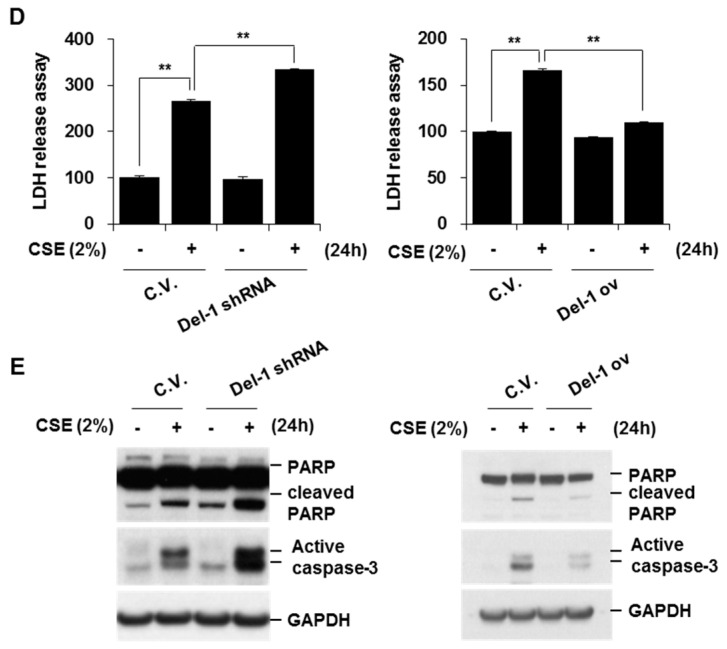
Del-1 suppressed CSE-induced IL-8 production and apoptosis in lung epithelial cells, without affecting aging marker expression or autophagy activation. HEK293T cells were transfected with pGIPZ−shCon, pGIPZ−shDel−1, Del1/pLOC, and pLOC, along with packaging vectors VSV−G and TRP, using lipofectamine reagent. A culture medium (1 mL) containing viral particles was added to BEAS-2B cells. Infected cells were used in experiments. (**A**) Total RNA was extracted, and quantitative real-time PCR for *Del−1* and *GAPDH* was performed. Data represent mean ± SD. ** *p* < 0.05. (**B**) Cells were treated with CSE (1%) for 24 h. The concentration of IL-8 in cell supernatants was measured using ELISA. Data represent mean ± SD. ** *p* < 0.05. (**C**) Cells were treated with CSE (1%) for the indicated periods. Cell lysates were subjected to Western blotting for p21, LC3B, and GAPDH. (**D**) Cells were treated with CSE (2%) for 24 h. Cell viability was determined using LDH release assay. Data represent mean ± SD. ** *p* < 0.05. (**E**) Cell lysates were subjected to Western blot analysis for PARP, active caspase-3, and GAPDH. Results are representative of three separate experiments. LDH, lactose dehydrogenase; ELISA, enzyme-linked immunosorbent assay.

To elucidate the role of Del-1 in COPD development, we next investigated the role of Del-1 in lung epithelial cells and macrophages. BEAS-2B cells were subjected to viral particle infections to establish Del-1 knockdown or overexpression in cells (Supplementary Figure S2). Del-1 expression in viral particle-infected BEAS-2B cells was assessed through real-time PCR. The Del-1 mRNA levels were significantly decreased by Del-1 shRNA and increased by Del-1 overexpression vector (Figure 3A). We tested the effects of Del-1 on CSE-induced IL-8 production; the expression of aging marker (p21); autophagy activation (LC3B); and cell death. CSE concentrations of 1% or less did not affect cell viability, so CSE at 1% was used in subsequent experiments. Del-1 knockdown amplified CSE (1%)-induced IL-8 production, a response mitigated by Del-1 overexpression (Figure 3B). Del-1 expression did not affect autophagy or cellular senescence in CSE (1%)-treated cells (Figure 3C). To test the effect of Del-1 on CSE-induced cell death, BEAS-2B cells were treated with CSE (2%), and a cell viability assay (LDH release) was performed. CSE (2%) induced cell death, with Del-1 knockdown exacerbating CSE-induced cell death. In contrast, Del-1 overexpression significantly inhibited cell death in CSE-treated cells (Figure 3D). Given that CSE is known to induce apoptosis—a form of programmed cell death—which is a critical factor in COPD pathogenesis [13], we examined the effect of Del-1 on CSE-induced apoptosis by measuring active caspase-3 and PARP cleavage. As demonstrated in the LDH release assay, Del-1 knockdown augmented CSE-induced apoptosis, which was effectively countered by Del-1 overexpression (Figure 3E). These results suggest that Del-1 plays a protective role against COPD development, potentially by suppressing neutrophilic inflammation and apoptotic cell death in epithelial cells.

### 2.4. Del-1 Suppressed LPS-Induced Pro-Inflammatory Chemokine/Cytokines in Macrophages

**Figure 4:**
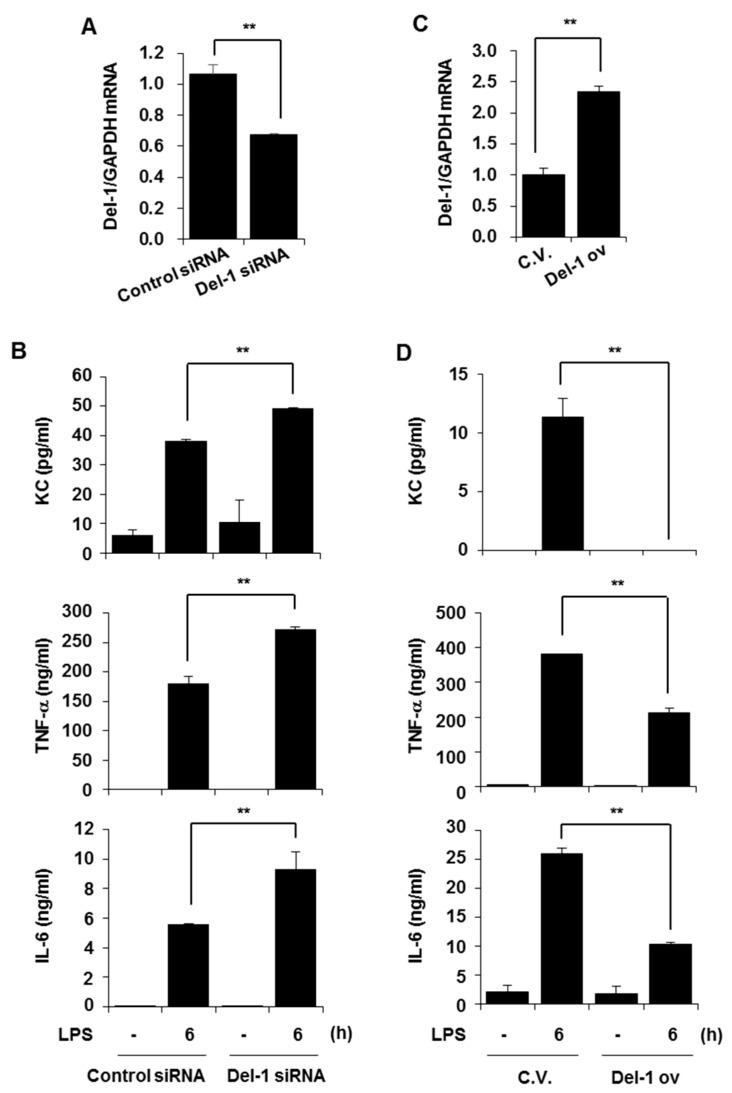
Del−1 suppressed LPS-induced pro-inflammatory chemokine/cytokines production in macrophages. (**A**–**D**) RAW264.7 cells were transiently transfected with control siRNA, Del−1 siRNA, pcDNA3.1 (control vector, C.V.), or Del-1 overexpression vector (Del−1 ov). After 48 h of transfection, cells were treated with LPS (100 ng/mL) for 6 h. (**A**,**C**) Total RNA was extracted and quantitative real-time PCR for *Del-1* and *GAPDH* was performed. (**B**,**D**) The concentrations of KC, TNF−α, and IL−6 in cell supernatants were measured using ELISA. Data represent mean ± SD. ** *p* < 0.05. Results are representative of three separate experiments.

Macrophages are recognized as crucial inflammatory cells in the pathogenesis of COPD and AE-COPD during bacterial infections [11]. Changes in Del-1 levels were observed in immune cells, including macrophages, in the lung tissues of smoker/COPD patients and CSE-instilled mice. Therefore, we next examined the role of Del-1 in macrophages. RAW264.7 cells were transiently transfected with control siRNA, Del-1 siRNA, control vector, or Del-1 overexpression plasmid vector. After 48 h of transfection, the cells were treated with LPS for 6 h. Del-1 expression levels were subsequently decreased in Del-1 siRNA-transfected cells (Figure 4A). The depletion of Del-1 significantly enhanced LPS-induced production of KC, TNF-α, and IL-6 (Figure 4B). In contrast, the overexpression of Del-1 inhibited LPS-induced chemokine/cytokine production (Figure 4C,D). These results underscore Del-1’s capacity to induce an anti-inflammatory response in macrophages as well as in lung epithelial cells. 

### 2.5. Del-1 Decreased the Levels of Phospho-p65, NF-kB-DNA Binding Affinity, and NF-κB Transcriptional Activity 

**Figure 5:**
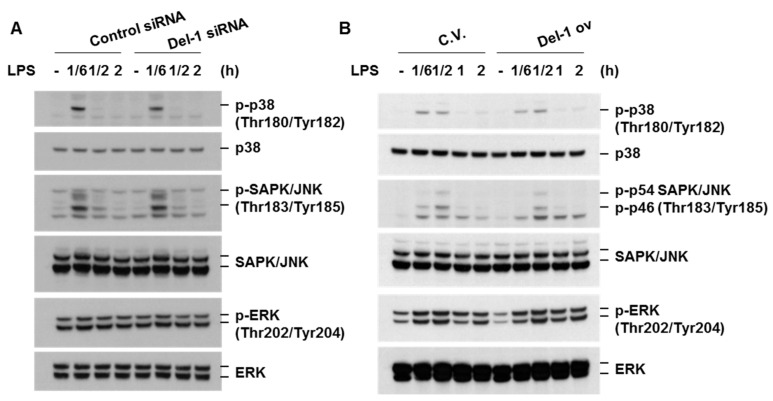
Del-1 did not affect the activation of MAP kinases. (**A**,**B**) RAW264.7 cells were transiently transfected with control siRNA, Del-1 siRNA, control vector (C.V.), or Del-1 overexpression vector (Del-1 ov). After 48 h of transfection, cells were treated with LPS (100 ng/mL) for the indicated times. Total cell extracts were subjected to Western blot analysis of phosphorylated p38 (p-p38), total p38 (p38), p-SAPK/JNK, total SAPK/JNK, p-ERK, and total ERK. Results are representative of three separate experiments.

We proceeded to investigate how Del-1 attenuates the inflammatory response. Initially, we examined the effect of Del-1 on LPS-induced activation of mitogen-activated protein (MAP) kinases, since the MAP kinase pathway is known to regulate pro-inflammatory cytokine production in response to Toll-like receptor stimulation [14]. Activation of the MAP kinase pathway was assessed through the analysis of phosphorylated MAP kinases (p38, SAPK/JNK, ERK) using immunoblotting. LPS-induced phosphorylation of p38, SAPK/JNK, and ERK remained unaffected by Del-1 expression (Figure 5A,B). These data suggest that Del-1-induced suppression of chemokine/cytokine production is not mediated through the inactivation of the MAP kinase pathway. 

**Figure 6:**
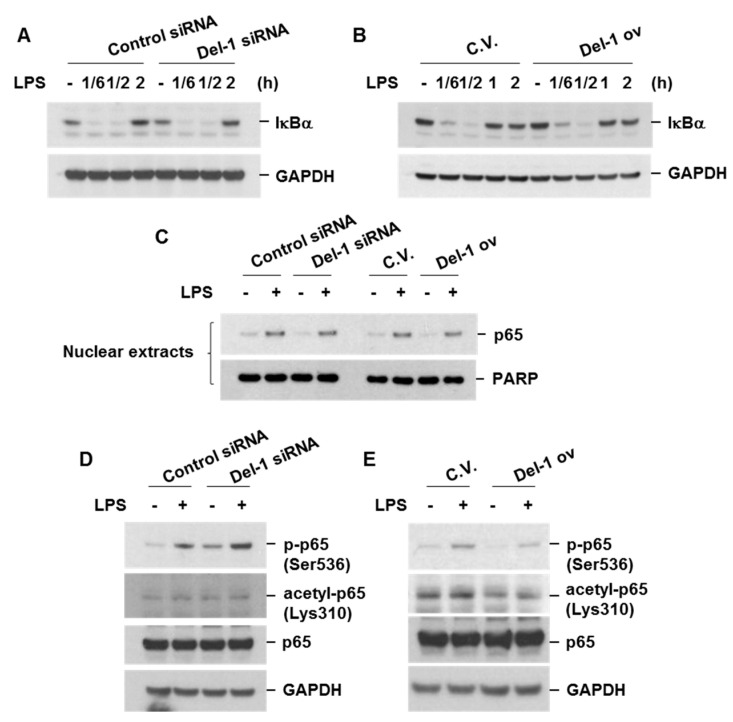

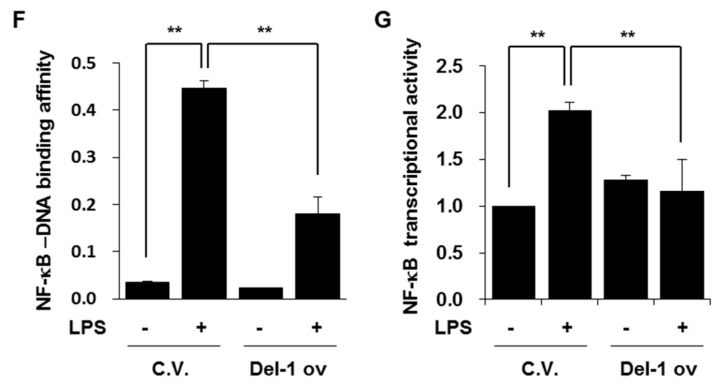
Del−1 did not affect the degradation of IκBα or the nuclear translocation of NF−κB p65, but decreased levels of phospho−p65, NF−κB−DNA binding affinity, and NF-κB transcriptional activity. (**A**,**B**) RAW264.7 cells were transiently transfected with control siRNA, Del−1 siRNA, control vector (C.V.), or Del−1 overexpression vector (Del−1 ov). After 48 h of transfection, cells were treated with LPS (100 ng/mL) for the indicated times. Total cell extracts were subjected to Western blot analysis of IκB and GAPDH. (**C**–**E**) Transfected cells were treated with LPS for 1 h. Nuclear extracts were subjected to Western blot analysis of p65 and PARP (**C**). Total cell lysates were subjected to Western blot analysis of p−p65 (Ser536), acetyl−p65 (Lys310), total p65 (p65), and GAPDH (**D**,**E**). (**F**) RAW264.7 cells were transfected with C.V. or Del-1 overexpression vector. After 48 h of transfection, cells were treated with LPS (100 ng/mL) for 4 h. Nuclear proteins were extracted, and the DNA binding activity of p65 in nuclear extracts was measured. (**G**) Cells were transfected with NF−κB reporter plasmid, C.V., or Del−1 overexpression vector. After 48 h of transfection, cells were treated with LPS (100 ng/mL) for 19 h. Luciferase activity was determined. Data represent mean ± SD. ** *p* < 0.05. Results are representative of three separate experiments.

NF-κB is a well-known transcription factor responsible for the production of various pro-inflammatory chemokines and cytokines in response to cellular stress. The activation of NF-κB involves the phosphorylation of IκBα by IκB kinases, followed by the subsequent degradation of IκBα and nuclear translocation of NF-κB [15]. Del-1 did not affect the degradation of IκBα or the nuclear translocation of NF-κB p65 in LPS-treated macrophages (Figure 6A–C). However, Del-1 knockdown enhanced the phosphorylation of the NF-κB subunit p65 at Ser536, whereas Del-1 overexpression repressed p65 phosphorylation, p65 acetylation at Lys310, NF-κB-DNA binding affinity, and the transcriptional activity of NF-κB (Figure 6D–G). These results indicate that the anti-inflammatory effect of Del-1 is mediated by the inhibition of p65 phosphorylation and acetylation. 

### 2.6. Activators of Nrf2 Increased Del-1 in Lung Epithelial Cells and Macrophages 

**Figure 7:**
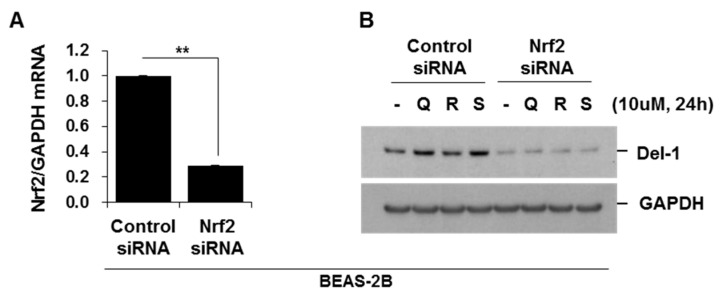

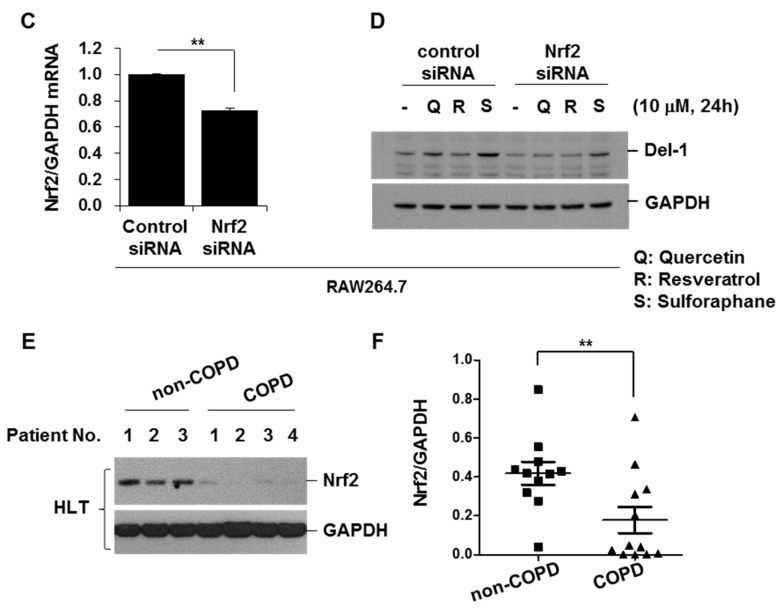
Activators of Nrf2 increased Del-1 in lung epithelial cells and macrophages. BEAS-2B (**A**,**B**) and RAW264.7 cells (**C**,**D**) were transiently transfected with control siRNA or Nrf2 siRNA. After 48 h of transfection, cells were treated with quercetin, resveratrol, or sulforaphane (10 μM) for 24 h. Total RNA was extracted, and quantitative real-time PCR for *Nrf2* and *GAPDH* was performed (**A**,**C**). Total cell lysates were subjected to Western blotting for Del-1 and GAPDH expression (**B**,**D**). Results are representative of three separate experiments. (**E**) The expression levels of Nrf2 in human lung tissues. Lung homogenates from non-COPD (smokers without emphysema, n = 11) and COPD patients (smokers with emphysema, n = 12) were subjected to Western blot analysis for Nrf2 and GAPDH. (**F**) Gel data were quantified using Scion image software Version 4.0. Data represent mean ± SE. ** *p* < 0.05.

Finally, we screened the potential activators of Del-1, which have the potential to attenuate COPD development. Nuclear factor erythroid 2-related factor 2 (Nrf2) is a transcription factor that plays a pivotal role in regulating cellular defenses against toxic and oxidative stress. Genetic ablation of Nrf2 has been shown to increase susceptibility to CSE-induced emphysema [16]. Moreover, Nrf2 exhibits protective effects against apoptosis and inflammation, similar to Del-1 [17]. The activators of Nrf2, such as quercetin, resveratrol, and sulforaphane, upregulated Del-1 in both lung epithelial cells and macrophages. Notably, the suppression of Nrf2 through siRNA resulted in a significant reduction of Del-1 upregulation in cells treated with quercetin, resveratrol, and sulforaphane (Figure 7A–D). Interestingly, just like Del-1, the expression of Nrf2 was observed to be lower in lung tissues from smoker/COPD patients when compared to smoker/non-COPD patients (Figure 7E,F). These results suggest that the expression of Del-1 is regulated by Nrf-2 activation.

## 3. Discussion

Del-1, a multifunctional molecule, maintains tissue homeostasis and resolves inflammation [8,10]. As a secreted protein, Del-1 regulates neutrophil recruitment and IL-17-driven inflammation. Furthermore, Del-1 resolves inflammation by promoting efferocytosis. With these anti-inflammatory properties and its high expression in lungs, the protective role of Del-1 against COPD development can be easily inferred. However, the role of Del-1 in COPD pathogenesis has not been fully understood. In this study, we demonstrated the protective effects of Del-1 on COPD development, and its underlying molecular mechanisms.

In our previous study, the protective effect of Del-1 in response to CSE was demonstrated through increased emphysema in *Del-1* KO mice [11]. Consistent with this, Del-1 levels were found to be lower in lung homogenates from patients with COPD than those from smokers without COPD. Interestingly, Del-1 expression was increased in smokers without COPD compared to non-smokers (data not shown). As the pathogenesis of COPD has been suggested to have stages of initiation, progression, and consolidation [18], we speculate that this phenomenon can be explained by the different stages of COPD. The relatively short-term exposure to noxious stimuli activates both pathogenic and endogenous defense mechanisms simultaneously, whereas long-term exposure depletes defense mechanisms to further increase the risk of COPD. Consistent with this, vascular endothelial growth factor (VEGF), which has a protective effect against the development of COPD, is also increased in moderate COPD, whereas VEGF is decreased in severe COPD [19,20]. Notably, in contrast to the decreased expression of Del-1 in human COPD patients, CSE instillation increased the expression of the Del-1 protein in the lung tissues of mice, as well as in the lung homogenates of smokers without COPD. Thus, the increased Del-1 expression in mice might be due to their relatively short-term exposure to CSE, whereas the decreased Del-1 expression in human lung tissues from COPD patients might reflect long-term exposure to smoking.

Given the more severe form of emphysema observed in *Del-1* KO mice, we investigated how Del-1 exerts protective effects against COPD. As the levels of Del-1 were changed in both lung epithelial cells and immune cells, including macrophages, we examined the role of Del-1 in both cell types. Knockdown of Del-1 in lung epithelial cells significantly increased CSE-induced IL-8 production and apoptosis. Conversely, Del-1 overexpression suppressed IL-8 production and apoptosis induced by CSE. IL-8 is well known as a potent neutrophil chemotactic factor, and COPD patients exhibit higher levels of IL-8 in their sputum and serum compared to control subjects [21,22]. Neutrophilic inflammation is a prominent feature of COPD, and apoptosis of airway epithelial cells has been reported to contribute to COPD pathogenesis [13]. Del-1 might prevent COPD development by suppressing neutrophilic inflammation and apoptosis in lung epithelial cells. 

The anti-inflammatory effects of Del-1 were also observed in macrophages. Del-1 markedly suppressed the production of neutrophilic chemokines and pro-inflammatory cytokines in LPS-treated macrophages. Our findings align with prior research [23], as the expression of Del-1 was found to be reduced in the alveolar lung epithelial cells of LPS-induced acute lung injury mice. Additionally, the overexpression of Del-1 mitigated LPS-induced lung injury by inhibiting inflammation and eosinophil recruitment [23]. Moreover, extracellular, soluble Del-1 suppresses apoptosis in endothelial cells when stimulated with etoposide or TNF-α/IFNγ through integrin binding [24]. These results suggest that the anti-inflammatory and anti-apoptotic effects of Del-1 are not specific to cell type or stimulus. 

The action mechanisms of Del-1 as an anti-inflammatory factor involve interaction with distinct integrins to limit leukocyte adhesion and migration, as well as the inhibition of IL-17 production [8]. In this study, we found that Del-1 did not affect the activation of MAP kinases, the degradation of IκBα, or the nuclear translocation of NF-κB subunit p65 in LPS-treated macrophages. However, while knockdown of Del-1 enhanced the phosphorylation of p65, overexpression of Del-1 suppressed the phosphorylation and acetylation of p65, resulting in a decrease in the NF-κB-DNA binding affinity and transcriptional activity of NF-κB. These results indicate that the anti-inflammatory effect of Del-1 is mediated by the inhibition of p65 phosphorylation and acetylation. It is worth noting that these findings are inconsistent with a previous study [24], which showed that recombinant Del-1 inhibits the production of macrophage migration inhibitory factor by attenuating the phosphorylation of IκBα and the subsequent translocation of NF-κB to the nucleus in LPS-treated RAW264.7 cells [25]. 

Interestingly, the expression of Del-1 progressively declines with aging. In old mice, Del-1 expression in periodontal tissue is severely diminished, and a deficiency of Del-1 accelerates aging phenotypes in young mice. However, the levels of Del-1 expression in lung tissues were not significantly correlated with patient age (Supplementary Figure S3) and Del-1 did not affect the CSE-induced expression of aging markers. 

Based on the regulatory effects of Del-1 on inflammation and apoptotic cell death, we assumed that Del-1 shares certain key functions with Nrf2, which also suppresses pro-inflammatory responses and apoptotic cell death [26,27]. Nrf2 diminishes oxidative stress, inflammation, and apoptosis in lung tissues. Furthermore, Nrf2 activators, such as quercetin, resveratrol, and sulforaphane, upregulated Del-1 in both lung epithelial cells and macrophages. Knockdown of Nrf2 significantly suppressed the upregulation of Del-1. Interestingly, similar to Del-1, Nrf2 expression was also lower in lung tissues from COPD patients compared to non-COPD patients. These results suggest that Del-1 expression might be regulated by Nrf-2 activation. 

Our study has some limitations. Firstly, while we confirmed the presence of intracellular Del-1 in lung epithelial cells and macrophages, we encountered challenges in distinguishing the functions of intracellular and extracellular Del-1. Secondly, although we demonstrated that Del-1 inhibits the phosphorylation and acetylation of NF-kB p65, the specific intracellular target of Del-1 could not be identified in this study. Further investigations are warranted to clarify these points.

## 4. Materials and Methods

### 4.1. Human Lung Tissues and Cells

Human lung tissues were obtained from lung resections performed during thoracic surgical procedures. After undergoing review and approval by the Seoul National University Hospital Institutional Review Board (SNUH IRB Number: H-1309-073-521), the specimens were collected. The normal human bronchial epithelial cell line (BEAS-2B) was purchased from American Type Culture Collection (ATCC, Manassas, VA, USA) and maintained in a defined keratinocyte serum-free medium (Life Technologies, Grand Island, NY, USA) at 37 °C under 5% CO_2_. Murine macrophage cells (RAW264.7) were obtained from the Korean Cell Line Bank (Seoul, Republic of Korea) and maintained in DMEM (Invitrogen, Carlsbad, CA, USA) containing 10% heat-inactivated FBS, 100 U/mL penicillin, and 100 mg/mL streptomycin at 37 °C under 5% CO_2_.

### 4.2. Reagents

Anti-poly (ADP-ribose) polymerase (PARP), anti-active caspase-3, anti-light chain 3B (LC3B), anti-phospho-p65 (Ser536) (p-p65), anti-acetyl-p65 (Lys310), anti-Nrf2, anti-IκBα, anti-p-p38 (Thr180/Tyr182), anti-p38, anti-p-p46/54 SAPK/JNK (Thr183/Tyr185), anti-SAPK/JNK, anti-p-ERK (Thr202/Tyr204), and anti-ERK antibodies were purchased from Cell Signaling Technology (Danvers, MA, USA). Anti-Del-1, anti-p21, anti-p65, and anti-glyceraldehyde 3-phosphate dehydrogenase (GAPDH) antibodies were obtained from Santa Cruz Biotechnology (Santa Cruz, CA, USA). Anti-8-hydroxy-2′-deoxyguanosine (8-OHdG) antibody was purchased from Bioss (Woburn, MA, USA). The anti-4-hydroxynonenal (4-HNE) antibody was purchased from Abcam (Boston, MA, USA). Lipopolysaccharide (LPS), quercetin, resveratrol, and sulforaphane were obtained from Sigma-Aldrich (St. Louis, MO, USA). 

### 4.3. Cigarette Smoke Extract (CSE) Preparation

Commercial cigarettes (THIS; 84 mm long with a diameter of 8 mm, purchased from Korea Tomorrow & Global Corp., Daejeon, Republic of Korea) were continuously smoked using a bottle system connected to a vacuum pump. Smoke from 20 cigarettes was bubbled through 60 mL of phosphate-buffered saline (PBS) (Life Technologies). The nicotine content was 0.65 mg and the tar content was 6.5 mg per cigarette. The puff duration was 22~24 s per cigarette. Any large insoluble particles in the resulting suspension were removed using a 0.22-μm filter [28].

### 4.4. Emphysema Mouse Model

Animal experiments were approved by the Institutional Animal Care and Use Committee (number 17-0134-C1A0(2), 09-0219-C1A0) of Seoul National University Hospital, Seoul, Korea. Female 8-week-old C57BL/6 mice were purchased from Koatech Laboratory Animal Company (Pyeongtaek, Republic of Korea). Female Del-1 knock-out (KO) mice with a C57BL/6 genetic background were kindly donated by Dr. Eun Young Choi (Asan Medical Center, Seoul, Republic of Korea). C57BL/6 WT and Del-1 KO mice were anesthetized, and either saline or 100 μL of CSE was intratracheally administered. CSE was administered once a week for eight weeks (WT saline, n = 4; WT CSE, n = 4; KO saline, n = 5; KO CSE, n = 5). The mice were euthanized the day after the last CSE instillation to isolate the lungs. 

### 4.5. Establishing a Stable Cell Line

pGIPZ-shCon, pGIPZ-shDel-1, Del-1/pLOC, and pLOC (Horizon Discovery Ltd., Waterbeach, UK) with packaging vectors VSV-G and TRP were transfected into HEK293T cells using lipofectamine reagent (Invitrogen), according to the manufacturer’s instructions. A culture medium (1 mL) containing viral particles was collected 72 h later and was added to BEAS-2B cells. Infected cells were sorted using a green fluorescent protein positivity assay to eliminate the uninfected cells. 

### 4.6. Transfection of Plasmid Vectors or siRNA

The control vector, the Del-1 expression vector (Sino Biological Inc., Houston, TX, USA), control siRNA, Del-1 siRNA (Thermo Fisher Scientific, Waltham, MA, USA), or Nrf2 siRNA (Santa Cruz Biotechnology) were transfected using the Neon Transfection System (Thermo Fisher Scientific), according to the manufacturer’s specifications. The cells were used in experiments after 48 h.

### 4.7. Lactate Dehydrogenase (LDH) Release Assay

Cytotoxicity was measured by the LDH release assay using a CytoTox-ONE homogeneous membrane integrity assay kit (Promega, Madison, WI, USA), according to the manufacturer’s instructions. 

### 4.8. Quantitative Real-Time Polymerase Chain Reaction (PCR)

Total RNA was isolated using an RNeasy kit (Qiagen, Hilden, Germany). cDNA was synthesized from 1 μg of total RNA using a Reverse Transcription System (Promega). PCR amplification was performed using a 2 × TaqMan Gene Expression Master Mix (Applied Biosystems, Foster City, CA, USA). Human *Nrf2* (Hs00975961_g1) and *GAPDH* probes (Hs99999905_m1) were obtained from Applied Biosystems. Power SYBR Green (Applied Biosystems) was used for PCR amplification. We used the following primers: human *Del-1* (forward: 5′-TCG AAG ACA TTG CAC TTT GC-3′, reverse: 5′-ACC CAG AGG CTC AGA ACA AC-3′); mouse *Del-1* (forward: 5′-CCT GTG AGA TAA GCG AAG-3′, reverse: 5′- GAG CTC GGT GAG TAG ATG-3′); mouse *Nrf2* (forward: 5′-CTC GCT GGA AAA AGA AGT G-3′, reverse: 5′-CCG TCC AGG AGT TCA GAG G-3′); mouse *GAPDH* (forward: 5′-ACG GCA AAT TCA ACG GCA CAG-3′, reverse: 5′-TGG GGG CAT CGG CAG AAG G-3′).

### 4.9. Protein Extraction and Western Blot Analysis

Total cellular extracts were prepared in 1× cell lysis buffer (Cell Signaling Technology). Frozen lung tissues (SNUH IRB Number: H-1309-073-521) were homogenized in tissue extraction buffer (Life Technologies) containing a protease inhibitor cocktail and phosphatase inhibitor mixture (Sigma-Aldrich). The cells were acclimatized for 5 min in ice-cold cytoplasmic extraction buffer (CEB) (10 mM Tris-HCl (pH 7.8), 10 mM KCl, 1.5 mM EDTA, and 0.5 mM DTT). The cells were then lysed on ice for 10 min in a 0.4% NP-40/CEB/protease inhibitor cocktail. The supernatants (cytoplasmic extracts) were collected through centrifugation at 3500 rpm for 5 min. The nuclear pellets were washed with CEB and suspended in a nuclear extraction buffer (NEB) consisting of 20 mM Tris-HCl (pH 7.8), 150 mM NaCl, 50 mM KCl, 1.5 mM EDTA, 5 mM DTT, and a 0.4% NP-40/protease inhibitor cocktail. The supernatant (nuclear extract) was collected through centrifugation at 13,000 rpm for 10 min. Protein concentrations were determined using the Bradford method (Bio-Rad, Hercules, CA, USA). Proteins were resolved using 4–12% sodium dodecyl sulfate-polyacrylamide gel electrophoresis (SDS-PAGE) and transferred to nitrocellulose membranes. Membranes were blocked with 5% skim milk blocking buffer containing primary antibodies for 1 h, before overnight incubation at 4 °C. The membranes were washed three times with washing buffer and incubated with secondary antibodies for 1 h. After successive washes, the membranes were developed using a SuperSignal West Pico Chemiluminescent kit (Thermo Fisher Scientific).

### 4.10. Cytokine Secretion Determination

Cytokine levels in the culture supernatants were determined using commercially available cytokine assay kits for human interleukin-8 (IL-8), mouse keratinocyte-derived chemokine (KC), mouse tumor necrosis factor-α (TNF-α), and mouse IL-6 (Bio-Plex Pro; Bio-Rad), according to the manufacturer’s instructions.

### 4.11. Immunohistochemistry

Lung tissues were fixed, paraffin-embedded, sectioned, and placed on slides using the Discovery XT automated immunohistochemistry stainer (Ventana Medical Systems Inc., Tucson, AZ, USA). Tissue sections were deparaffinized and rehydrated. Cell conditioning 1 standard (pH 8.4 buffer containing Tris/Borate/EDTA) was used for antigen retrieval. The sections were then incubated with an anti-Del-1, anti-8-OHdG, or anti-4-HNE antibody for 32 min at 37 °C, washed, and incubated with a secondary antibody for 20 min at 37 °C. After successive washes, slides were incubated with 3,3-diaminobenzidine and H_2_O_2_ substrate for 8 min at 37 °C, followed by hematoxylin and bluing reagent counterstaining. Stained cells were observed under a microscope (EVOS XL Core Cell Imaging System, Thermo Fisher Scientific).

### 4.12. NF-κB p65 Transcription Factor Assay

NF-κB p65 DNA-binding activity in nuclear extracts was detected using a non-radioactive and sensitive ELISA-based assay kit (Abcam, Boston, MA, USA), following the manufacturer’s instructions. Briefly, a double-stranded DNA sequence containing the NF-κB response element was immobilized on the bottom of the wells of a 96-well plate. NF-κB p65 was contained in nuclear extracts bound to the NF-κB response element, and was detected using an anti-NF-κB p65 antibody. A secondary antibody conjugated to horseradish peroxidase was added to provide a colorimetric readout at 450 nm. 

### 4.13. NF-κB Luciferase Activity Assay

Cells cultured in 35 mm dishes were transfected with the NF-κB reporter or control plasmids using a Neon transfection system (Invitrogen), according to the manufacturer’s specifications. Luciferase activity was determined using a Luciferase Assay Kit (Promega).

### 4.14. Statistical Analysis

Statistical analysis was performed using GraphPad Prism software Version 9 (San Diego, CA, USA). Data were analyzed using a two-tailed unpaired *t*-test or the Mann–Whitney U-test, as appropriate, to determine statistical significance. Data from in vitro cell experiments are presented as mean ± SD. Data from experiments using human lung tissues and mice are expressed as mean ± SE. Statistical significance was set at *p* < 0.05.

## 5. Conclusions

This study demonstrates the protective role of Del-1 against COPD pathogenesis. This protective effect is, in part, mediated by its anti-inflammatory and anti-apoptotic actions. The restoration of down-regulated Del-1 presents a potential strategy for the prevention and treatment of COPD.

## Data Availability

The data within this article will be shared on request to the corresponding author.

## Supplementary Materials

The following supporting information can be downloaded at: https://www.mdpi.com/article/10.3390/ijms25041955/s1.
