# Supplementary material for: Del-1 Plays a Protective Role against COPD Development by Inhibiting Inflammation and Apoptosis
Source: Int J Mol Sci. 2024 Feb 6;25(4):1955. doi: 10.3390/ijms25041955 (PMC10888117; doi:10.3390/ijms25041955)
Supplement: Supplementary file 1 [file ijms-25-01955-s001.zip › ijms-2785094-supplementary.pdf]

Supplementary Figures

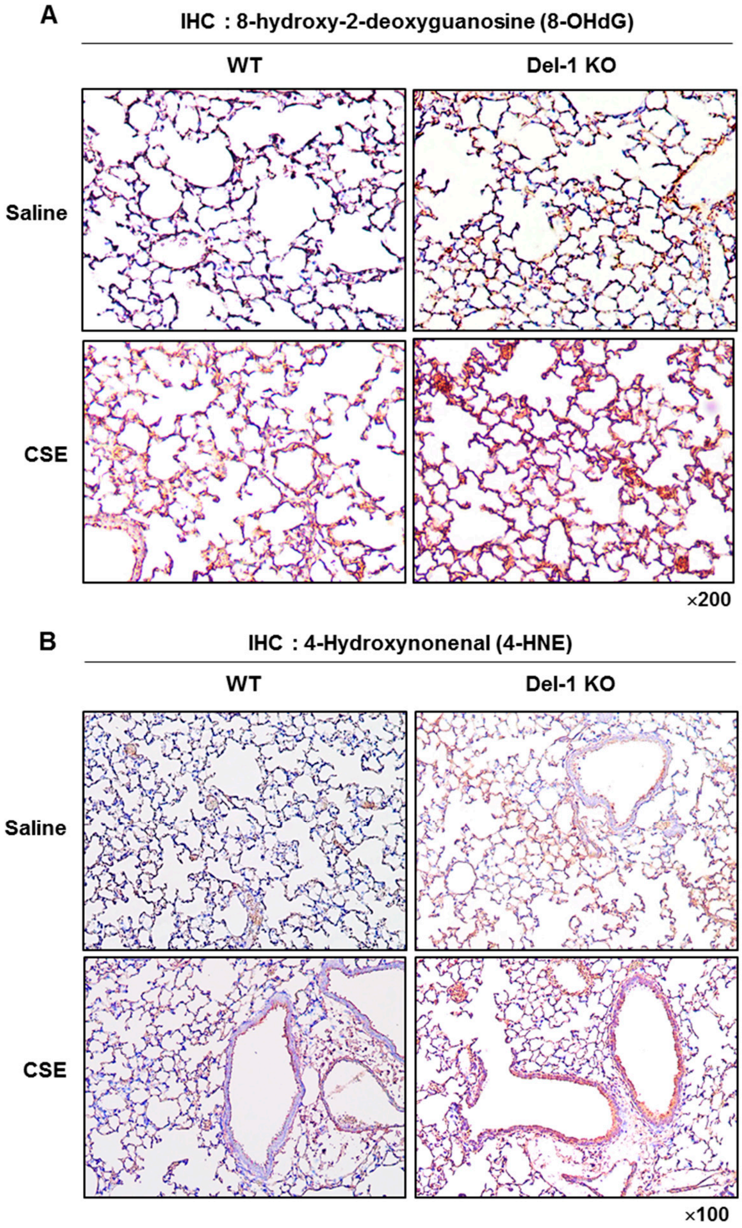

Supplementary Figure S1. CSE-induced expression of 8-OHdG and 4-HNE was higher in those of Del-1 KO mice than in the lung tissues of WT mice. C57BL/6 WT and Del-1 KO mice were intratracheally instilled with saline or CSE as described in Materials and Methods. 8-OHdG (A) and 4-HNE (B) immunohistochemistry of lung tissues from saline- or CSE-treated mice.

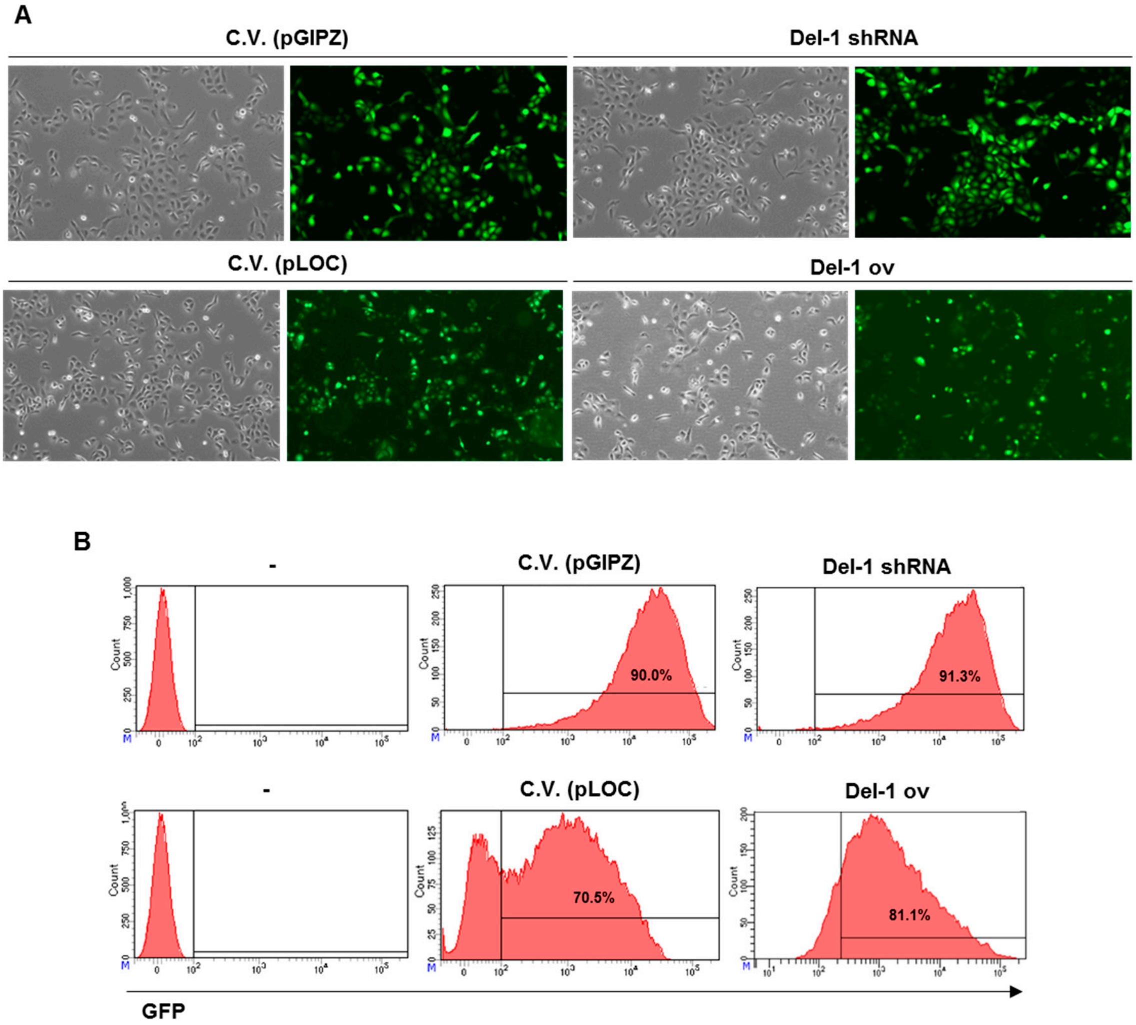

Supplementary Figure S2. Establishing a stable cell line. pGIPZ-shCon, pGIPZ-shDel-1, Del-1/pLOC, and pLOC vectors with packaging vectors VSV-G and TRP were transfected into HEK293T cells using lipofectamine reagent according to the manufacturer's instructions. A culture medium (1 mL) containing viral particles was collected 72 h later and was added to BEAS-2B cells. Infected cells were sorted using a green fluorescent protein (GFP) positivity assay to eliminate the uninfected cells. (A) Infected cells were analyzed using ECLIPSE TE300 (Nikon) fluorescence microscope. (B) GFP-positive cells was determined by flow cytometry. Results are shown as MFI histograms.

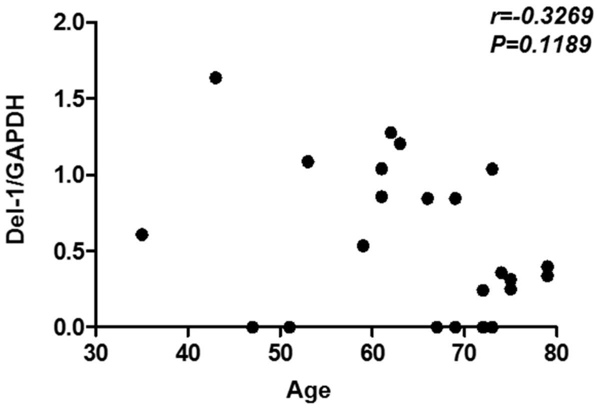

Supplementary Figure S3. Del-1 expression was not correlated with age. Pearson correlation coefficient, r, was calculated for the expression level of Del-1 of human lung tissues (y-axis) and age (x-axis) of 24 subjects including non-COPD (n = 12) and COPD patients (n = 12).
